# Physicochemical characterisation, immunogenicity and protective efficacy of a lead streptococcal vaccine: progress towards Phase I trial

**DOI:** 10.1038/s41598-017-14157-7

**Published:** 2017-10-23

**Authors:** Manisha Pandey, Jessica Powell, Ainslie Calcutt, Mehfuz Zaman, Zachary N. Phillips, Mei Fong Ho, Michael R. Batzloff, Michael F. Good

**Affiliations:** Institute for Glycomics, Griffith University, Queensland, 4222 Australia

## Abstract

Globally, group A streptococcal infections are responsible for over 500,000 deaths per year. A safe vaccine that does not induce autoimmune pathology and that affords coverage for most GAS serotypes is highly desired. We have previously demonstrated that a vaccine based on the conserved M-protein epitope, J8 was safe and immunogenic in a pilot Phase I study. We subsequently improved vaccine efficacy by incorporation of a B-cell epitope from the IL-8 protease, SpyCEP, which protected IL-8 and enhanced neutrophil ingress to the site of infection. We have now substituted the carrier protein, diphtheria toxoid with its superior analogue, CRM197 which provides better immunogenicity and is widely used in licenced human vaccines. The new vaccine was compared with the DT conjugate vaccine to confirm that these modifications have not altered the physicochemical properties of the vaccine. This vaccine, when tested in an animal model of GAS infection, demonstrated significant reduction in systemic and local GAS burden, with comparable efficacy to the DT conjugate vaccine. The vaccine was shown to be equally effective in the presence of human plasma and in the presence of pre-existing DT-specific antibodies, thus minimising concerns regarding its potential efficacy in humans.

## Introduction

Infections with *Streptococcus pyogenes* (group A *Streptococcus*; GAS) remain a major public health problem in resource-limited settings, constituting an important cause of morbidity and mortality. GAS is a versatile pathogen capable of producing a spectrum of human diseases ranging from mild infections such as pharyngitis and impetigo to invasive diseases such as cellulitis, toxic shock syndrome and necrotising fasciitis. The burden of invasive GAS disease is alarming with at least 663,000 new cases and 163,000 deaths each year^1^. Moreover, repeated streptococcal infection may lead to the development of the post-infection sequelae of rheumatic fever, rheumatic heart disease and acute post streptococcal glomerulonephritis. Furthermore, autoimmune reactions may produce a number of neuropsychiatric disorders, including Sydenham’s chorea, obsessive-compulsive disorder and PANDAS syndrome^2^. Altogether, there are more than 500,000 deaths due to GAS per year globally and treatment for GAS disease costs several billion dollars in the United States alone^1^. An effective GAS vaccine is therefore highly desirable to prevent primary GAS infections and to reduce mortality and morbidity.

A number of approaches have been adopted to develop a GAS vaccine. While, some target the M-protein (N- and C-terminal portions), others are based on non-M-protein antigens and include streptococcal C5a peptidase, streptococcal carbohydrate, streptococcal fibronectin binding proteins, cysteine proteases, streptococcal pyrogenic exotoxins and streptococcal pilli (as reviewed^3^). The vaccine, based on peptides derived from the N-terminal domain of the M-protein, was found to be immunogenic and safe in clinical trials without adverse effects^4,5^; however, the vaccine is expected to have limited coverage in developing countries and there are concerns that it may trigger a shift in serotype prevalence^6^. Another lead vaccine based on an M-protein conserved region minimal epitope (J8) has been tested for its safety in the Lewis Rat model for valvulitis in parallel with a rabbit toxicology study. These studies demonstrated that the J8-DT vaccine (J8 conjugated to diphtheria toxoid) did not induce abnormal pathology^7^. The vaccine has also been tested in a pilot study and shown to be immunogenic in humans with no serious adverse events reported in the study (manuscript in preparation). The high sequence conservation of the vaccine epitope suggests that it has the potential for wide coverage.

In a number of preclinical studies, we have demonstrated that J8 conjugated to diphtheria toxoid (J8-DT) is efficacious in protection in animal models against multiple GAS strains^8^. However, we further demonstrated that the vaccine has diminished efficacy against hyper-virulent CovR/S mutant GAS strains, due to their augmented ability to degrade IL-8 thus preventing neutrophil chemotaxis. To rectify that, a 20-mer B-cell epitope (S2) from the streptococcal IL-8 protease, SpyCEP, was incorporated with J8-DT. This resulted in a combination vaccine (J8-DT + S2-DT) that was highly effective in protection against CovR/S mutant GAS strains^9^. We demonstrated that both J8 and S2 were poorly immunogenic (cryptic) to humans and mice following infection^9,10^. However, they were highly immunogenic as peptide vaccines and able to induce protective responses.

We are now progressing this vaccine towards human clinical trials. However, with a view to developing a consistent product, the vaccine has undergone modifications. The carrier protein, diphtheria toxoid (DT) is replaced with its chemically defined genetically modified analogue, CRM197 (henceforth referred to as CRM). CRM is an enzymatically inactive and nontoxic form of diphtheria toxin that contains a single amino acid substitution (G52E)^11^. Unlike DT, CRM does not require detoxification with formaldehyde and homogenous preparations of purified antigen can be readily obtained. CRM is a precisely defined protein, consistent from batch to batch. It is licenced for human use in several efficacious conjugate vaccines^12,13^. A further modification is that the S2 peptide has been reformed with lysine residues (K4S2) to improve its solubility in aqueous solution. The present study reports the comparison of CRM with DT for the preparation of the peptide conjugate vaccine J8-CRM + K4S2-CRM (henceforth referred to as MJ8CombiVax), its characterisation, immunogenicity and protective efficacy in a murine challenge model. Finally, the study also addresses elements that are critical to vaccine efficacy in humans, including the effect of prior exposure to DT and the effect of human plasma (which is known to contain proteins capable of binding to the M-protein) on vaccine efficacy.

## Results

### Design and assessment of K4S2

We previously demonstrated that a 20-mer linear B-cell epitope from SpyCEP, S2, was immunogenic in mice as well as in humans. We also demonstrated that this epitope was the principal target for anti-SpyCEP antibodies that could protect IL-8 from SpyCEP -mediated proteolysis^9^. However, the solubility of S2 in aqueous solution was low, which was likely due to the presence of hydrophobic amino acids such as phenylalanine (F) in its structure (Supp Table 1). Therefore, to increase the solubility of S2 in aqueous solution, a number of S2 derivatives (namely K2S2K2, K4S2 and S2K4) were designed with hydrophilic lysine residues added to its side chain. Addition of four lysine residues to the N-terminus, or C-terminus, or 2 lysine residues added to each of the N and C-termini of S2 resulted in increased hydrophilicity (hydropathy index  −2.05) compared to the parent S2 peptide (hydropathy index  −1.70). These modifications significantly enhanced the solubility of all the three S2 derivatives in aqueous solution in comparison to the parent peptide, S2.

**Figure 1:**
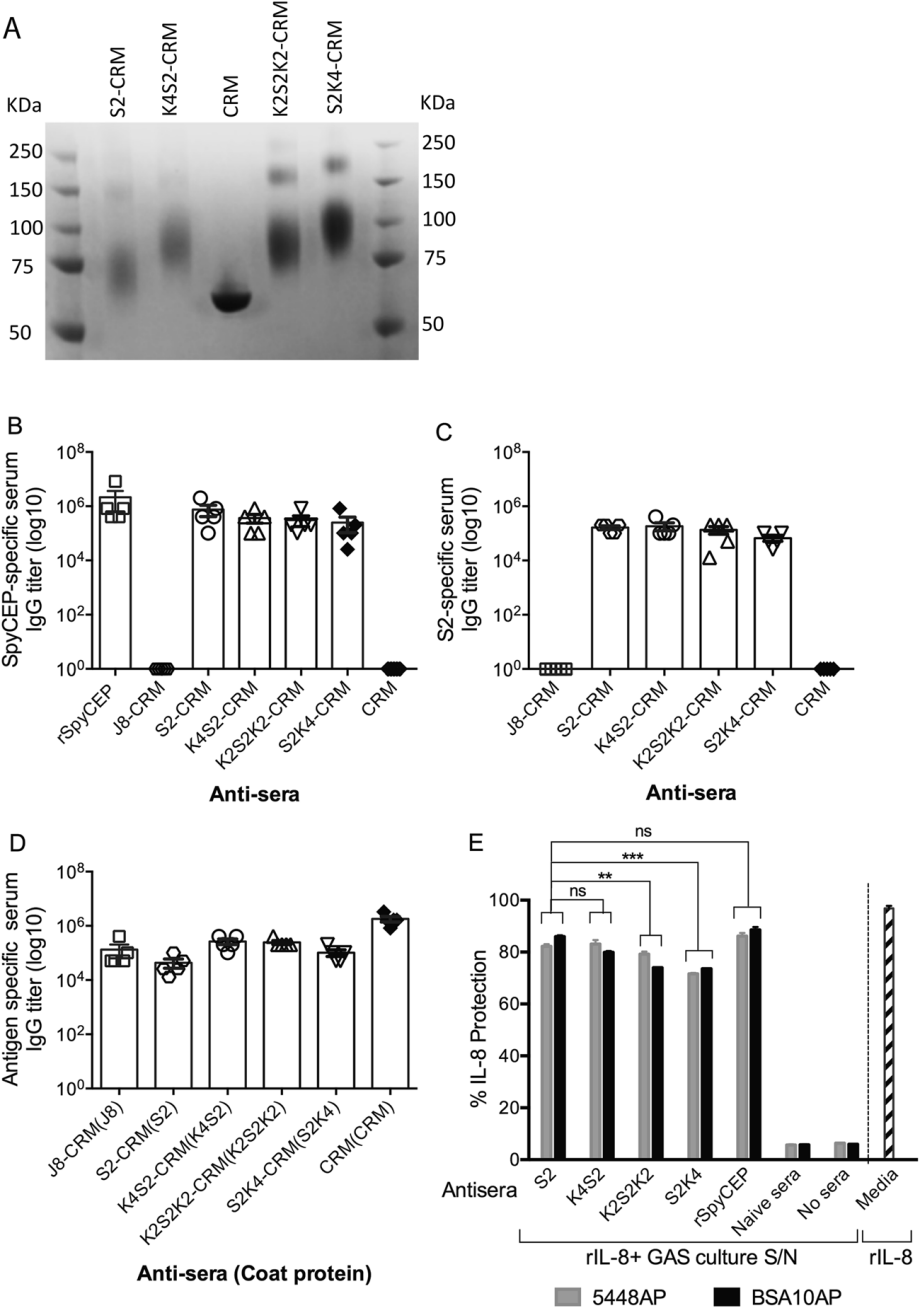
SDS-PAGE profile of S2 derivatives-CRM197 conjugates. (**A**) To enhance the solubility of SpyCEP epitope S2 in aqueous solution, lysine residues were added to S2 at either N-or C-termini, or at both ends. The resulting three S2 derivatives, as well as the parent S2 peptide, were conjugated to CRM197 (CRM) and characterised by SDS-PAGE. CRM alone was used as a control. The protein conjugates were resolved by 4–15% poly acrylamide gels and visualised using commassie blue stain. A cropped image of the gel is presented (see Sup Fig. 1 for full image). (**B**–**D**) Immunogenicity of S2 derivatives. The immunogenicity of each S2 derivative-CRM conjugate was assessed *in vivo*. BALB/c mice (female, 4–6 weeks, n = 5/group) were immunised intramuscularly (IM) with S2 or S2 derivative-CRM conjugate, adjuvanted with Alum and antibody titers to rSpyCEP (**B**) or to parent S2 peptide (**C**) were assessed. Antibody titers to each immunogen were also investigated and are shown (**D**). CRM alone was used to assess cross-reactivity to other antigens (**B** and **C**) and to assess CRM-specific IgG titers (**D**). Data are for 5 individual mice with the line showing mean ± SEM. (**E**) IL-8 protection assay. To assess the SpyCEP neutralising ability of S2 derivatives antiserum, an IL-8 protection assay were performed. Cell-free culture supernatants from various CovR/S MT GAS strains were co-incubated with 50 ng/mL of rIL-8 and either 1 in 50 dilution of normal mouse, rSpyCEP or S2-derivative antiserum for 16 h at 37°C. Wells with normal serum were used as a negative control. As an internal control for IL-8 degradation, rIL-8 with media alone was also included. Data for each bar are Mean ± SEM. Statistical analysis was performed using one-way ANOVA with Tukey’s post-hoc test to determine significance between the groups. ns, p > 0.05; **p < 0.01 and ***p < 0.001.

The three S2 derivatives, with cysteine, were then conjugated to CRM using Sulfo-EMCS as described previously^14^. All three conjugates demonstrated a similar profile on SDS-PAGE (Fig. 1A, Sup Fig. 1), which was comparable to that of the S2-CRM conjugate. To select the best S2 derivative, the immunogenicity of each conjugate was tested in BALB/c mice. Mice immunised with each of the conjugates, adjuvanted in Alum, demonstrated comparable immunogenicity, with anti-SpyCEP or anti-S2 specific IgG titers of ≥ 10^5^ (Fig. 1B and C). The titers to the immunizing conjugate were also similar (≥10^5^) (Fig. 1D). Finally, to compare the functionality of each S2 derivative-CRM conjugate, an IL-8 protection assay was performed. Cell-free culture supernatants from various GAS strains were co-incubated with rIL-8 and either a 1:50 dilution of normal mouse serum or SpyCEP- or S2-specific antisera for 16 h at 37 °C. Sera from mice immunized with all three derivatives of S2 protected IL-8 significantly better than the control serum; however, the K4S2 antisera demonstrated the highest protection, comparable to the protection offered by S2- or rSpyCEP-specific antisera (Fig. 1E).

### Characterisation of the CRM-conjugated vaccine and its comparison with the DT-conjugated vaccine

#### Conjugation characteristics of DT- and CRM- conjugate vaccines

The vaccine peptides (J8 and K4S2) that were individually conjugated to CRM were compared with DT- conjugated peptides. SDS-PAGE analysis demonstrated a similar conjugation profile for the corresponding peptide conjugates (Sup Fig. 2A). Amino acid analysis confirmed a similar conjugation ratio of J8 to DT or to CRM, with a loading ratio of 1:8–12 (where one molecule of DT/CRM conjugated to 8–12 J8 units). The ratio for S2 on DT/CRM was in the range of 1:7–13 and for K4S2 was 1:6–14. Of note was the observation that CRM or CRM conjugates formed a sharper band following gel electrophoresis compared to DT and DT conjugates (Sup Fig. 2A).

#### Adsorption of vaccine conjugate onto Alhydrogel (Alum)

*In vitro* studies were performed to compare the adsorption of DT- and CRM- conjugated vaccines onto Alum. Known amounts of vaccine conjugates (J8 or K4S2, conjugated to DT or CRM) were incubated with Alum in 1:1 ratio (v/v). The conjugate-Alum formulations were then incubated at various temperatures (RT, 4° or 37°) for 0 h, 1 h or overnight and the amount of unbound protein was measured in the supernatant. The data demonstrated that all the DT or CRM conjugated vaccines were readily adsorbed onto Alum. All corresponding conjugate pairs J8-DT/J8-CRM (Sup Fig. 2B), K4S2-DT/K4S2-CRM (Sup Fig. 2C), or the combination vaccine pair J8-DT + K4S2-DT/J8-CRM + K4S2-CRM (Sup Fig. 2D) demonstrated comparable adsorption onto Alum with less than 5% residual (unbound) antigen in the supernatant.

### Biological efficacy of vaccine conjugates

#### Assessment of immunogenicity of DT- and CRM- conjugated vaccines

**Figure 2:**
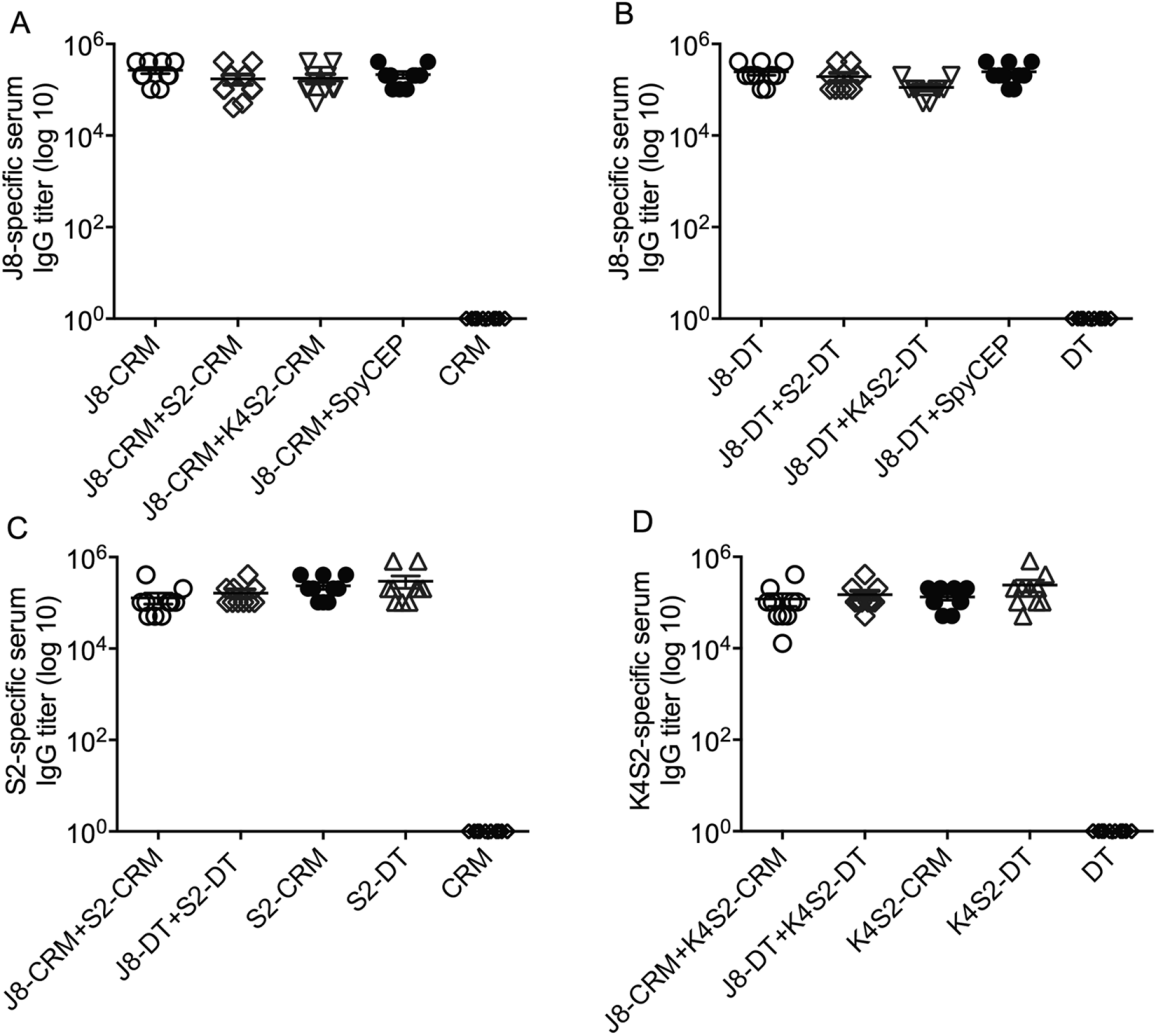
Immunogenicity of DT and CRM conjugated peptides. Cohorts of BALB/c mice (4–6 weeks, n = 10/group) were immunised intramuscularly with peptide-DT or peptide-CRM conjugates on day 0, 21 and 28. The mice received either individual peptide-DT/CRM conjugates or admixed preparations of two peptide-DT/CRM conjugates. One week post-last boost, the mice were bled via the submandibular vein. J8-specific IgG titers of individual or combination peptide-CRM conjugates (**A**) and peptide-DT conjugates (**B**) are shown. Similarly S2-specific IgG titers of S2 or J8 + S2 conjugated to CRM and DT (**C**) and K4S2-specific IgG titers of K4S2 or J8 + K4S2 conjugated to CRM and DT are shown (**D**). CRM or DT alone was used as control.

Next, the immunogenicities of CRM- or DT- conjugated vaccines were assessed in a murine model. Mice were immunised with the combination vaccine formulations (J8-DT + S2-DT/Alum and J8-DT + K4S2-DT/Alum or J8-CRM + S2-CRM/Alum and J8-CRM + K4S2-CRM/Alum) as well as individual vaccine formulations (J8-DT/Alum, S2-DT/Alum or K4S2-DT/Alum). Serum samples were collected at defined time-points and ELISAs performed to determine antibody titers. All vaccine formulations with J8, induced comparable J8-specific IgG titers (>10^5^) (Fig. 2A and B). These data also demonstrated that within the limitations of this experiment, the presence of other peptide conjugates in the formulation, such as S2-DT, K4S2-DT or rSpyCEP did not affect the immunogenicity of J8. Immunization with DT or CRM alone did not result in cross-reactive antibodies to J8 (Fig. 2A and B). Both the DT- and CRM- conjugates with S2 or K4S2 generated similar self-titers, confirming their comparable immunogenicities (Fig. 2D). Finally both DT and CRM combination conjugate vaccines also generated comparable antigen-specific titers (Fig. 2C and D).

#### Binding of vaccine antisera to GAS isolates

**Figure 3:**
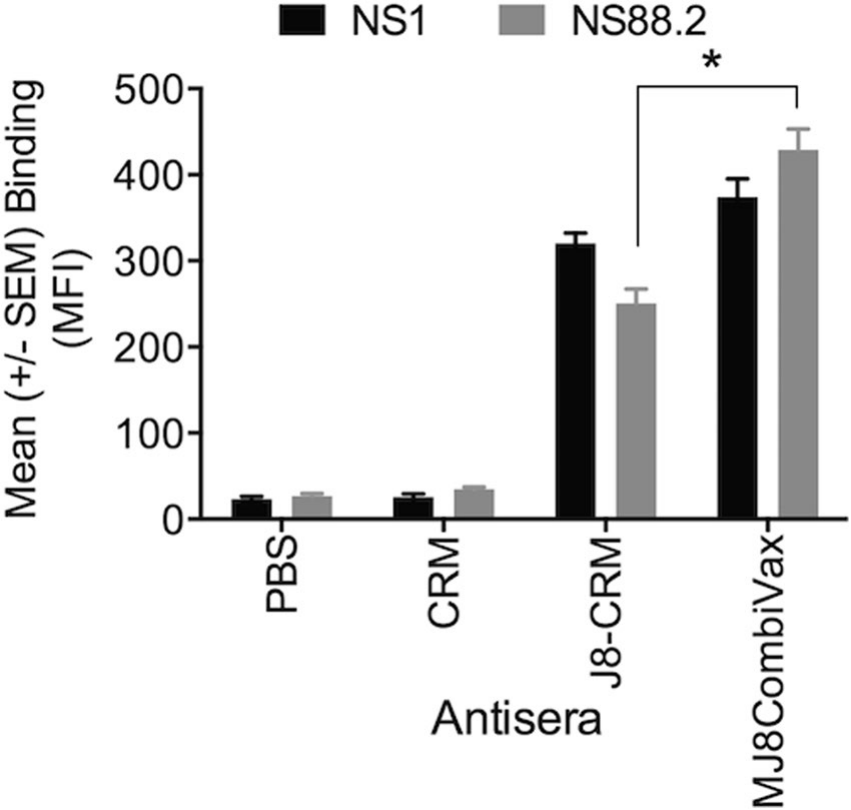
Binding of vaccine antisera to GAS. To assess the binding of vaccine antisera (J8-CRM or MJ8CombiVax) to the surface of various GAS isolates, flow cytometry was performed. The assay measured the binding of vaccine antibody through FITC conjugated secondary IgG. Binding of J8-CRM or MJ8CombiVax antibodies to GAS NS1 (CovR/S WT) and GAS NS88.2 (CovR/S MT) are shown as mean fluorescence intensity (MFI). The antisera from CRM or PBS immunised mice were used as controls. Data for each bar are Mean ± SEM. Significance between the binding efficiency of different vaccine antisera to the same GAS strain was determined using a student t-test. *p < 0.05.

We used flow cytometry to assess the binding of CRM-conjugated vaccine antibodies to the surface of GAS, expressing different *emm* types. Incubation of J8-CRM and J8-CRM + K4S2-CRM (MJ8CombiVax) antiserum with NS1 GAS (CovR/S Wild Type [WT]) and NS88.2 GAS (CovR/S Mutant Type [MT]) strains, revealed a distinction in antibody binding efficiency of these vaccine antisera. The binding of J8-CRM antisera to NS1 and NS88.2 were comparable as was the case with MJ8CombiVax antisera (Fig. 3). However, MJ8CombiVax antisera bound significantly better to NS88.2 than did J8-CRM antisera. This could be attributed to the high level expression of S2/SpyCEP on the surface of NS88.2 GAS, which is known for CovR/S mutant GAS strains. These data thus suggest that the use of the combination vaccine leads to antibody recognition of more than one antigen on GAS resulting in a higher binding efficiency. The binding of CRM antisera was insignificant.

### Immunological assessment of carrier priming on the anti-peptide response

**Figure 4:**
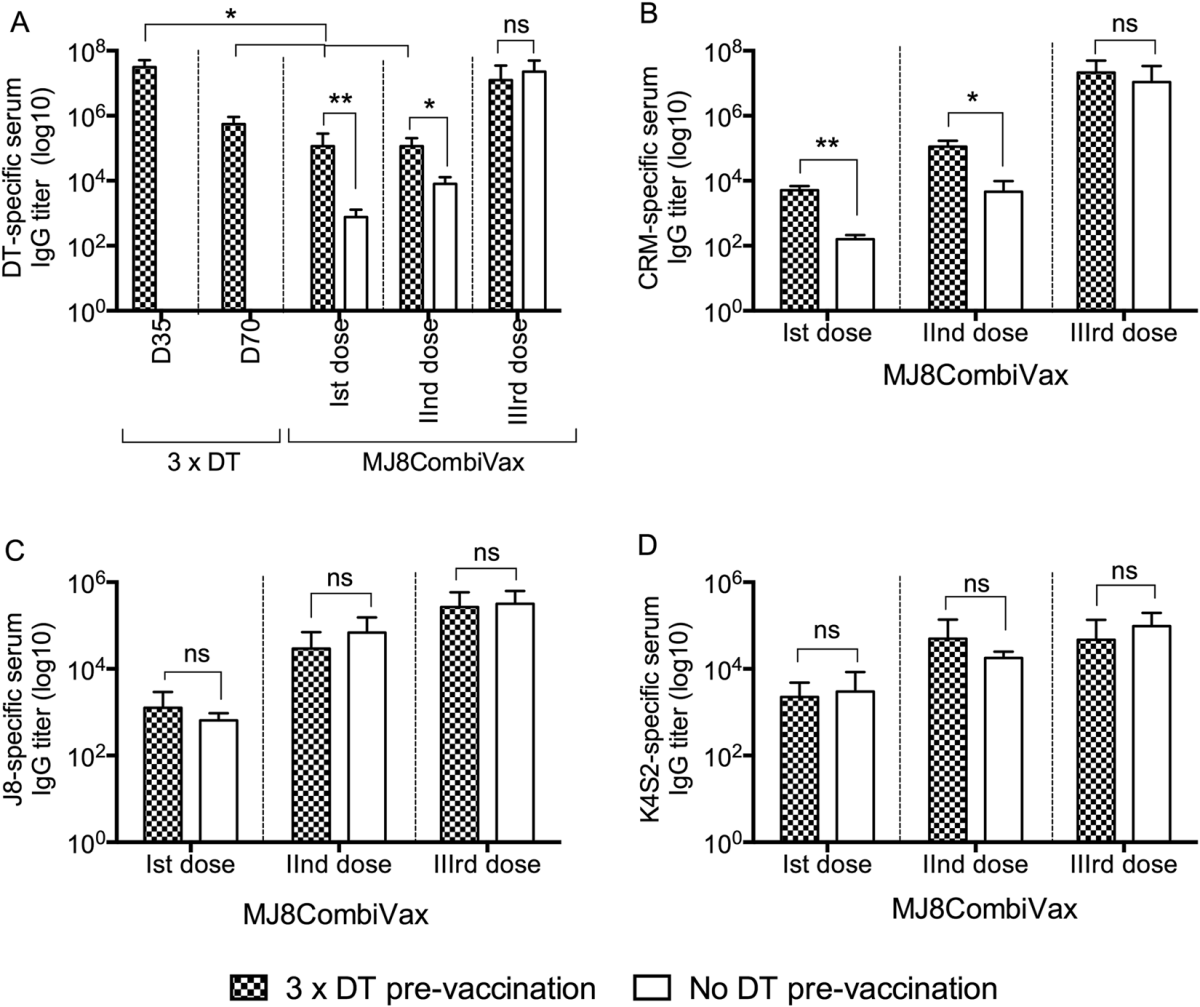
Effect of pre-existing DT antibodies on immunogenicity of CRM conjugated vaccines. (**A**) Cohorts of BALB/c mice were immunised intramuscularly with three doses of DT/Alum at 30 μg each on day 0, 21 and 28. Serum samples were collected one-week post-last boost and then following a rest period of four weeks to measure DT-specific serum IgG. This was followed by three intramuscular immunisations (D0, D21 and D28) with MJ8CombiVax. In parallel, a cohort of naive BALB/c mice (without DT pre-immunisations) was also vaccinated with MJ8CombiVax. Both the DT primed and naive mice were tested for peptides and carrier specific IgG post immunisation with MJ8CombiVax. The mice were bled post each dose (d20, d27 and d35) and antigen specific IgG titers measured. The IgG titers for both the cohorts post each immunisation for DT (**A**), CRM (**B**), J8 (**C**) and K4S2 (**D**) are shown. Statistical analysis was performed using student t-test to determine significance between the two groups at each time points. ns, p > 0.05; *p < 0.05; **p < 0.01.

To investigate if the pre-existing DT antibodies (the common scenario in a population following childhood immunisation) would affect the immunogenicity of the CRM conjugated vaccine, additional immunisation studies were undertaken. BALB/c mice, previously immunised with three doses of DT, were rested for four weeks prior to their vaccination with three standard doses of MJ8CombiVax. Post rest, a significant decline (p < 0.05) in DT-specific IgG was noted, which was regained post 3xMJ8CombiVax administrations (Fig. 4A). We observed that following the1^st^ and 2^nd^ doses of MJ8CombiVax, DT primed mice had significantly higher levels of DT and CRM-specific IgG in comparison to naïve mice (p < 0.05 to p < 0.01) (Fig. 4A and B). However, following three doses of MJ8CombiVax the levels of DT and CRM-specific IgG in both cohorts were comparable (Fig. 4A and B). Furthermore, the J8- and K4S2-specific IgG in DT-primed and naïve cohorts, post each immunisation, were comparable (Fig. 4C and D). The data thus demonstrate that the vaccination with MJ8CombiVax results in boosting of DT/CRM IgG in DT-primed mice. Most importantly it shows that the immunogenicities of both J8 and K4S2 remain unaffected by pre-existing DT-specific IgG.

### Protective efficacy of CRM conjugate vaccines against CovR/S WT and MT GAS strains

**Figure 5:**
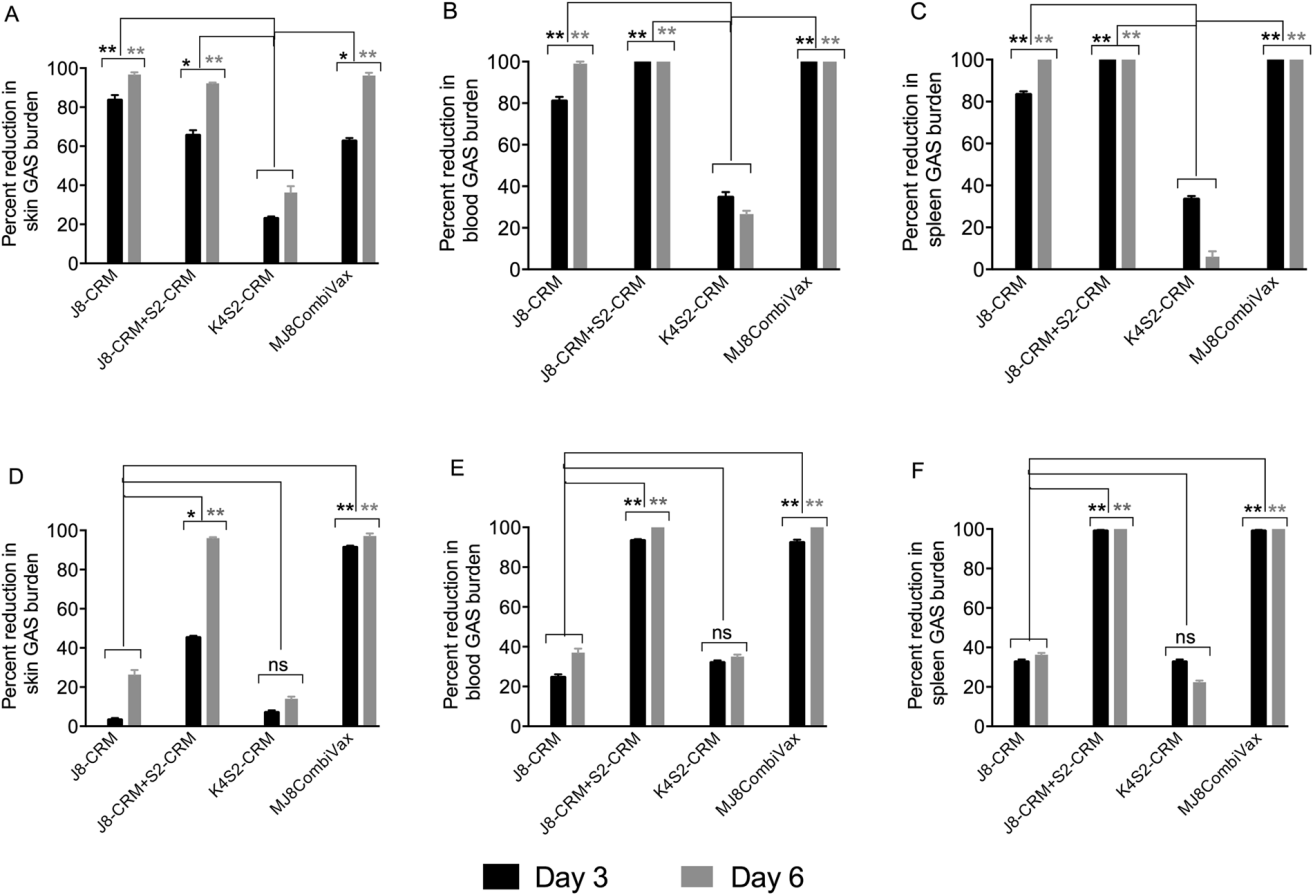
Protective efficacy of CRM-conjugated combination vaccines against CovR/S WT and hypervirulent GAS. Cohorts of BALB/c mice were immunised intramuscularly with either MJ8-CombiVax or individual vaccine components. An additional cohort immunised with J8-CRM + S2-CRM was also included for comparison. Two weeks following last immunisation, the mice were challenged via the skin route of infection with two different GAS strains, NS1 (**A**–**C**), or NS88.2 (**D**–**F**). On days 3 and 6 post infection, 5 mice/group were sacrificed and samples were collected to determine bacterial burden. Percent reduction in skin, blood and spleen bacterial burden in comparison to the naïve challenge control (n = 5/group) was calculated and is shown as mean ± SEM. The mean bacterial burdens for NS1 GAS in control mice were 5115000 and 41850 (skin); 34440 and 22200 (blood); 522240 and 59450 (spleen) on days 3 and 6 respectively. For NS88.2 GAS, the mean bacterial burdens in control mice were 6695000 and 2045000 (skin); 77750 and 52460 (blood); 890700 and 731400 (spleen) on days 3 and 6 respectively. Statistical analysis was performed using one-way ANOVA with Tukey’s multiple comparison tests to compare each group with every other group at each time point. ns, p > 0.05; *p < 0.05; **p < 0.01.

We previously demonstrated that the combination vaccine comprising J8-DT + S2-DT protects mice from pyoderma and bacteremia following challenge with CovR/S MT GAS strains^9^. In this study we investigated if the S2 derivative, K4S2, would be functionally comparable to S2 and if MJ8CombiVax will be equally efficacious in protection against CovR/S WT and MT strains. Mice were vaccinated with MJ8CombiVax or with individual vaccine components. Mice vaccinated with J8-CRM + S2-CRM were also included as controls. Following skin challenge with NS1 GAS, skin, blood and spleen samples were analysed for bacterial burden. Our data demonstrated that both the J8-CRM as well as MJ8CombiVax were efficacious in protecting mice against pyoderma and systemic infection on day 3 post-infection. The reduction in skin and blood/spleen bacterial burden was further improved by day 6, reaching more than a 95% reduction, which was comparable to parent combo vaccine J8-CRM + S2-CRM (Fig. 5A–C). We noted that protection offered by K4S2-CRM alone was limited (20–30%).

Challenge with NS88.2 GAS demonstrated that J8-CRM alone was ineffectual in protection against CovR/S MT strain. In these cohorts, both the local (Fig. 5D) and systemic bacterial burdens (Fig. 5E–F) were still more than 60% compared to the control mice. Nevertheless, MJ8CombiVax was very effective in protection against this mutant strain and resulted in >95% reduction in local and systemic bacterial load by day 6 (Fig. 5D–F).

### Functionality of vaccine antisera in the presence of human plasma

**Figure 6:**
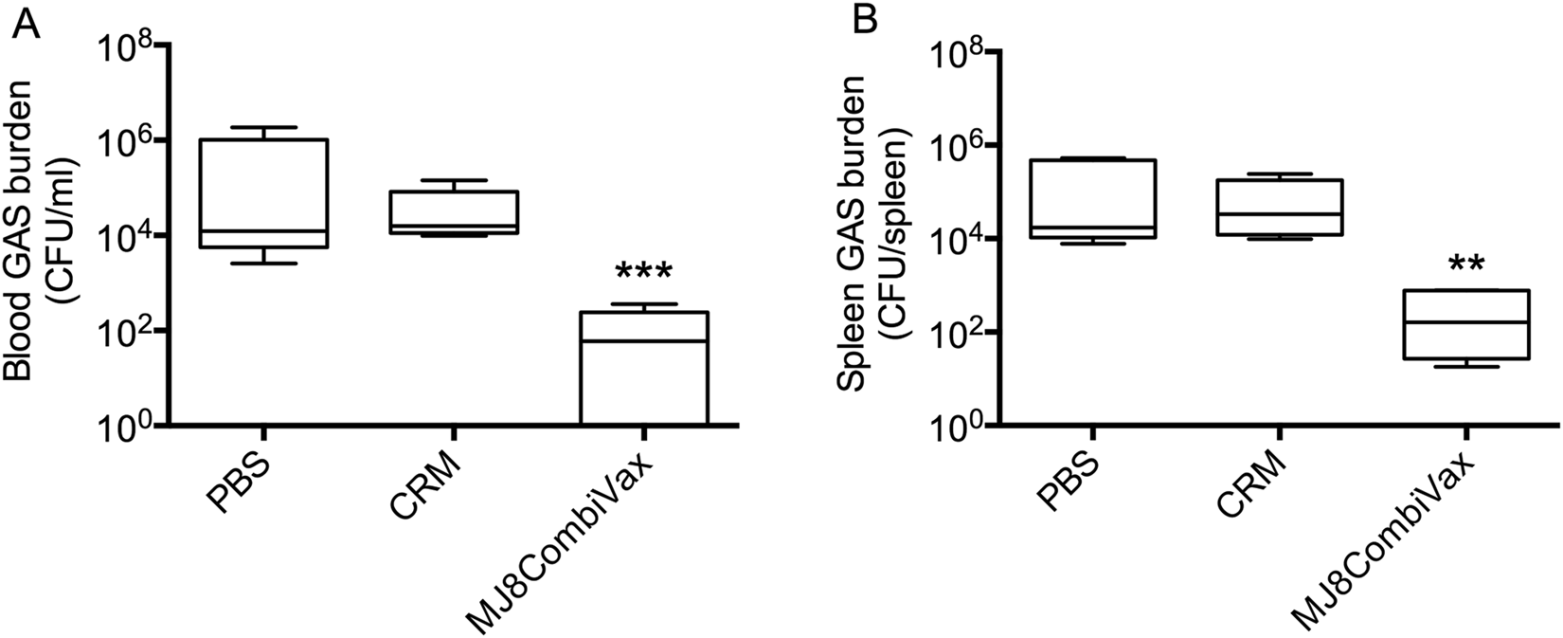
Protective efficacy of vaccine antisera in the presence of human plasma. To assess if the presence of human plasma will inhibit the opsonic ability of vaccine antisera, GAS pre-incubation-challenge experiments were undertaken. GAS NS88.2 strain was co-incubated with antisera from either MJ8CombiVax-, CRM- or PBS-vaccinated mice and human plasma (mixed 1:1) for 1 h at 4 °C. The serum/plasma mix was removed by washing the bacterial pellet and the resuspended bacterial inoculum was injected intraperitoneally into SCID mice. Post 48 h of bacterial challenge, mice were culled and bacterial burden in blood and spleen were assessed. The results are shown as box and whisker plot where the line in the box is indicating the median, the box extremities indicating the upper and lower quartiles and the whiskers showing minimum to the maximum values. One-way ANOVA with Tukey’s post-hoc method was utilised to calculate significance between the control and test groups. **p < 0.01 and ***p < 0.001.

Finally to demonstrate the functionality of the vaccine in the presence of human plasma components *in vivo*, phagocytosis assays were performed under different incubation conditions. Initially we tested if human plasma proteins would inhibit the binding of vaccine antisera to 2031 (*emm1*) GAS. Our data demonstrated that preincubation of GAS with human plasma inhibited the binding of vaccine antisera to GAS (Sup Fig. 3A). However, if GAS were co-incubated with vaccine antisera and plasma, the inhibitory effect of plasma proteins was negated (Sup Fig. 3B). The functionality of vaccine antibodies in the presence of human plasma was then confirmed in a SCID mouse protection assay. The NS88.2 GAS (CovR/S MT) was co-incubated with vaccine antisera in the presence of human plasma prior to intraperitoneal (IP) administration into naïve SCID mice. Our data demonstrated that the presence of human plasma did not inhibit the killing ability of vaccine antibodies. In the presence of antibodies from J8 and IL-8 protease-neutralising antibodies from K4S2, GAS were killed and mice were protected significantly better (p < 0.01 to 0.001) than the control cohorts (Fig. 6A and B). The GAS incubated with CRM or PBS antisera, in the presence of plasma, did not show any reduction in local or systemic bacterial burden in SCID mice (Fig. 6A and B).

## Discussion

A conserved epitope vaccine against group A *Streptococcus* would be a major advance in reducing GAS carriage and GAS related disease by providing a broad coverage of circulating strains. Ideally, it would also target clinically significant strains associated with life threatening complications such as invasive infections and post-streptococcal sequelae. Strategies to develop a broad-strain coverage GAS vaccine have included the design of type-specific multivalent M-protein vaccine constructs containing epitopes from the amino terminal type-specific regions of several different M-protein serotypes^15–18^, vaccine candidates based on the conserved C-region of the M-protein^14,19,20^ and vaccine approaches incorporating both type-specific and conserved region determinants^21,22^. Here, we show that a combination of two vaccines using the commonly used licensed carrier protein, CRM197 (a derivative of diphtheria toxin), with defined and highly conserved epitopes from two virulence factors (the M-protein and SpyCEP) is effective in preventing skin and deep tissue infection caused by hyper-virulent CovR/S mutant, as well as wild type, *Streptococci*. We demonstrate the mechanism of immunity and show that the presence of human plasma proteins will not impede vaccine efficacy. Further, we show that pre-existing immunity to DT will not affect vaccine efficacy.

We previously demonstrated that the J8-DT vaccine induces strain-transcending immunity to common GAS strains^14,23,24^. However, hyper-virulent organisms upregulate virulence factors including the IL-8 protease, SpyCEP, which impede J8 vaccine-induced immunity by blocking neutrophil ingress. This led us to develop the second component of the vaccine and augment J8 vaccination by incorporating a recombinant or synthetic fragment of SpyCEP^8,9^. We recently demonstrated that the combination vaccine facilitates neutrophil accumulation at the site of infection and protects against local infection and invasive GAS disease with hyper-virulent organisms^8,9,25^. The two component-combination vaccine has an enhanced safety profile as it comprises only minimal epitopes (12 amino acids from the conserved region of GAS M-protein; safety previously demonstrated^7^;) and a 20 contiguous amino acid epitope from SpyCEP, which has not been linked to any post streptococcal sequelae.

In this study we reformulated and tested the vaccine with an enzymatically inactive and nontoxic form of DT – CRM197. Both the CRM and DT conjugated vaccines, were compared in parallel in a range of physicochemical and immunological studies. We found that both the carrier proteins conjugated to the antigenic peptides, J8 and S2, in a similar manner. This was confirmed by amino acid analysis where both peptide conjugates demonstrated similar protein: peptide conjugation ratios. The SDS-PAGE data suggested a possible dimerization of some of the carrier proteins (DT or CRM). However, it did not have any notable effect on the immunogenicity of the vaccine. The adsorption of both peptide conjugates onto Alum was also very similar (>95%). We made three derivative of S2 to improve its solubility in an aqueous solution. All three S2 derivatives, with lysine residues, demonstrated a similar SDS-PAGE profile when conjugated to CRM and comparable immunogenicities. However, the IL-8 protection ability of the K4S2 conjugate was superior. The selected K4S2 peptide, whether conjugated to DT or CRM, and in combination with J8-DT or J8-CRM, demonstrated comparable immunogenicity to the corresponding peptides. Also, vaccine administration via intramuscular route in this study demonstrated comparable immunogenicity to subcutaneous vaccine administrations^9^.

The affinity of vaccine antibody for GAS was tested in a flow cytometry based assay. J8-CRM or MJ8CombiVax murine antisera demonstrated comparable binding to NS1 GAS. However, MJ8CombiVax antiserum bound significantly better to NS88.2 GAS compared to J8-CRM antisera. NS88.2 is a CovR/S mutant GAS that is known to express/shed high level of the IL-8 protease, SpyCEP. We believe that the higher binding of MJ8CombiVax antiserum to NS88.2 is due to the binding of S2 antibodies present in the serum. We have previously demonstrated that S2 antibodies bind better to CovR/S mutant GAS strains^9^ and also in this study we show that S2 and K4S2 antibodies share antigen specificity.

Pre-existing immunity to a vaccine carrier protein has been reported to inhibit the immune response against antigens conjugated to the same carrier by a process called carrier induced epitopic suppression (CIES)^26^. This is a critical issue for the development of vaccines where conjugation to a carrier protein is required to enhance the immunogenicity of poorly immunogenic or hapten antigens. Likewise, it was a concern for us, as the carrier protein DT/CRM, in our candidate vaccine, is also a licenced and commonly used vaccine. A small number of studies have explored the phenomenon of CIES in humans but the results are inconclusive. While a few studies showed reduced antigen-specific antibody titers after carrier priming, others showed either no effect or increased titers^27,28^. A study focussing on B-cells as antigen-presenting cells, concluded that hapten-carrier conjugates were presented by B-cells in pre-immunised animals whereas it was presented by dendritic cells in naïve animals^29^. The difference in antigen presentation was suggested to lead to the induction of Th2 versus Th1 cells. Here we report that priming with DT followed by 3 doses of MJ8CombiVax resulted in boosting of the DT-specific IgG response; however, no effect on the J8 or K4S2-specific responses was observed. These data support the previous findings by Pecetta *et al*.^30^ where carrier priming with DT was shown not to supress the immunogenicity of CRM conjugates.

In the current study our lead vaccine, J8-DT + S2-DT, was modified to enhance its solubility while maintaining its immunogenicity with a better-defined and superior conjugation partner. Therefore, it was of utmost importance to ensure that vaccine efficacy remains unaffected. By conducting a series of GAS challenge experiments, we have demonstrated that the advanced combination vaccine J8-CRM + K4S2-CRM (MJ8CombiVax) is equally efficacious as its predecessor J8-DT + S2-DT and it has a better safety prospective in humans. By using NS1 (CovR/S WT) GAS we again confirmed that J8-CRM although effective against common GAS strains, needs the synergistic action of S2/K4S2 to block SpyCEP activity and allow neutrophil chemotaxis, to be efficacious against CovR/S MT strains such as NS88.2.

We have shown that vaccine-specific antibodies bind to the surface M-protein of GAS, which, in the presence of neutrophils result in opsonophagocytic killing of GAS^14^. We also demonstrated that in neutropenic mice or mice infected with CovR/S mutant GAS strains (where IL-8 is degraded and neutrophil influx to the infection site is inhibited), vaccine efficacy is compromised. The combination vaccine, J8-DT + S2-DT, rectifies this issue and by neutralising the IL-8 protease, SpyCEP, protects IL-8 and thus the process of chemotaxis^9^.

An earlier study by Sandin *et al*.^31^, argued that human plasma proteins such as fibrinogen and albumin would inhibit antibody binding under normal physiological conditions by specifically binding to the B- and C-repeats of the M-protein. Pre-incubation with human serum albumin (HSA) was shown to inhibit binding of antibodies to the C-repeat region^31^. In our studies by utilising two different experimental protocols, we demonstrated that inhibition of antibody binding to GAS is dependent on the reaction conditions. When 2031 (*emm1*) GAS were incubated with human plasma, prior to addition of vaccine antiserum, a significant reduction in vaccine antiserum binding to GAS was observed. However, when human plasma and vaccine antiserum were admixed (1:1 ratio) prior to addition to GAS, no inhibition in binding to GAS was noted. We believe that pre-incubation with plasma partially blocks the C-repeat binding sites whereas co-incubation with vaccine antiserum and human plasma results in competitive binding. In this situation the vaccine antibodies, by virtue of their high affinity for the C-repeat region of M-protein, bind to GAS and the presence of plasma proteins cannot prevent that. The co-incubation protocol employed here has the advantage that it reflects the human physiological situation where vaccinated individuals will possess both the plasma proteins and vaccine-induced antibodies prior to their encounter with GAS. The killing ability of vaccine antibodies was further confirmed by transferring GAS, pre-incubated with vaccine antisera, into SCID mice. These data further confirmed that not only were the vaccine antibodies able to bind to GAS (both CovR/S WT and MT type), but that they were also functionally competent leading to killing of GAS.

One potential caveat of this study is the use of a mouse model. Since GAS is a human-specific pathogen, it rarely shows natural virulence for mice and therefore requires serial passaging through mice to enhance its virulence. Moreover, mice do not respond to GAS superantigens (sAg) and antibodies to sAg may protect mice from GAS challenge^32^. While these represent limitations to the use of mouse models for studying GAS pathogenesis, the data presented in the manuscript can be extrapolated to propose that vaccination with MJ8CombiVax, by reducing bacterial burden, will minimise/eliminate the effects of GAS sepsis and sAg-induced pathology in infected individuals.

The data presented in this study provide a framework for a human trial of MJ8CombiVax and suggest that the vaccine will be efficacious in humans and protect against virulent streptococci of multiple serotypes.

## Methods

### Human and animals ethics

Human blood was collected from healthy adult volunteers. All protocols were approved by the Griffith University Human Research Ethics Committee, GU ref No: BDD/01/15/HREC. The study was carried out in accordance with the National Health and Medical Research Council (NHMRC) of Australia. An informed consent was obtained from all subjects. BALB/c mice (female, 4–6 weeks old) were sourced from Animal Resource Centre (ARC, Perth, Western Australia). All protocols were approved by Griffith University’s Animal Ethics Committee (GU-AEC) in accordance with the National Health and Medical Research Council (NHMRC) of Australia guidelines. Methods were chosen to minimise pain and distress to the mice. Animals were observed daily by trained animal care staff. Mice were terminated using a CO_2_ inhalation chamber.

### Bacterial strains and growth conditions

GAS isolates obtained from various sources were utilised in the study. *S pyogenes* 2031 (*emm1*) is an isolate from Prague reference center; BSA10 (*emm124*) and NS1 (*emm100*) are clinical isolates from the Northern Territory of Australia. All these strains were obtained from the Menzies School of Health Research, Darwin. The isolates were passaged through mice and made streptomycin-resistant (200 μg/mL) by continually replating them with increasing concentrations of streptomycin. 5448AP (*emm1*) and NS88.2 (*emm*98.1), CovR/S mutant GAS strains, were obtained from the Walker laboratory (University of Queensland, Brisbane, Australia). The isolates were grown in Todd-Hewitt broth (Oxoid, Adelaide) supplemented with 1% (wt/vol) neopeptone (Difco). Challenge inoculum were prepared as previously described^9^.

### Peptides and carrier conjugation to peptides

Peptide K4S2 and other S2 derivatives were designed using the protein bioinformatics tool, GPMAW lite. Their hydropathy index was analysed using the Kyte-Doolittle scale^33^ and accordingly lysine residues were added to S2 to decrease its hydrophobicity. The peptides J8 and the S2 derivatives including K4S2 peptides (with cysteine at the N-terminus) were commercially sourced (China peptides Co.) and conjugated to DT or CRM (Pfenex Inc) using N-(ε-maleimidocaproyloxy) sulfosuccinimide ester (Sulfo-EMCS; Thermo Fisher Scientific) following the manufacturer’s protocol. SDS-PAGE (4–15% gel) analysis was performed to confirm the conjugations. To determine the peptide-protein ratios, amino acid analysis was performed at the Australian Proteome Analysis Facility (APAF, Brisbane, Australia).

### Adsorption of DT and CRM conjugates on to Alum

DT and CRM conjugated peptides (J8 and K4S2 individually or admixed), at known concentrations, were mixed with Alum (Alhydrogel; Brenntag Biosector) in 1:1 (v/v) ratio. The conjugate-Alum mix was then incubated under various conditions including 0 h RT; 1 h 4 °C; 1 h 37 °C; O/N 4 °C and O/N 37 °C. Following incubation, the formulation was spun at 2000 RPM for 15 minutes and supernatant collected. The unbound peptide in the supernatant was quantified using Micro BCA assay (ThermoFisher Scientific, USA).

### Immunisation and detection of murine antibodies

BALB/c mice (female, 4–6 weeks) were immunised intramuscularly on day 0, 21 and 28 with 30 μg of peptide-DT or peptide-CRM conjugates formulated in alhydrogel (Alum, Brenntag Biosector, Denmark) in 1:1 ratio (v/v), as previously described^25^. For the combination vaccine, 30 μg of each conjugate was admixed in a ratio of 1:1 by weight and formulated in Alum as previously described^9^. Control mice received saline with adjuvant alone. For carrier priming experiments, mice were immunised three times with DT and then rested for 4 weeks before they received three immunisations with MJ8CombiVax. A parallel cohort of naïve mice was also vaccinated with MJ8CombiVax. In all experiments, one-week post last immunisation mice were bled via submandibular vein and serum isolated. The serum samples were stored at −20 °C until used. ELISA was used to determine murine Ag-specific IgG antibody titers as previously described^34^. Goat anti-mouse IgG-HRP (Bio-Rad laboratories) was used to determine Ab titers. Titre was defined as the highest dilution that gave an optical density (OD) reading of more than three standard deviations (SD) above the mean OD of control wells containing normal mouse sera at the same dilution^34^.

### IL-8 protection assay

The assay was conducted as previously described^9^. Briefly GAS strains 5448AP and BSA10AP were grown to stationary phase. The cell free culture supernatants were co-incubated with recombinant IL-8 and a 1 in 50 dilution of either rSpyCEP, S2, S2 derivatives, or PBS antisera for 16 h at 37 °C. IL-8 with media alone was used as a positive control. Uncleaved IL-8 was measured using a quantikine ELISA kit (R & D systems), and neutralisation of chemokine cleaving activity due to test antiserum was calculated and compared to the controls (IL-8 with normal serum/no serum/media alone).

### Flow cytometry

To assess the binding of vaccine antisera to GAS, flow cytometry was performed as previously described^9^. Briefly Fc blocker (monoclonal antibody 2.4G2) was used to block non-specific binding sites and pooled antiserum from test and control mice, at a dilution of 1:100, was used to stain the bacterial cell surface. FITC conjugated anti-mouse IgG (diluted 1:200) was used as a secondary antibody and samples were analysed using CyAn ADP Analyzer (Beckman Coulter, Inc.). The antisera from CRM or PBS immunised mice was used as control. For selected experiments, GAS organisms were incubated with test or control sera in the presence or absence of normal human plasma and the influence of human plasma proteins in the inhibition of vaccine antisera binding was investigated.

### Skin challenge and sample collection

Two-three weeks following immunisation, mice were challenged via the skin route of infection as previously described^8^. GAS strains NS1 (CovR/S Wild Type, WT) and NS88.2 (CovR/S Mutant Type, MT) were used in this study. Post-challenge the mice were monitored closely for their welfare. On days 3 and 6 post GAS challenge, five mice were culled and blood and tissue samples collected for assessment of bacterial burden. To determine CFU in skin, the entire section of skin lesion was homogenised and 10-fold serial dilutions were plated on blood agar plates. To assess systemic infection, 10-fold serial dilution of spleen homogenate and blood samples were plated and CFU enumerated after the plates were incubated overnight at 37 °C.

### Pre-incubation-challenge experiments

To assess the functionality of vaccine antibodies in the presence of human plasma, SCID mice protection studies were performed. Pooled human plasma from healthy volunteers was used in the study. Pre-determined CFU of GAS were co-incubated with vaccine/control antisera with human plasma (1:1) for 1 h at 4 °C with gentle shaking. Following incubation, the GAS suspension (in 400 μL final volume) was injected intraperitoneally into naïve SCID mice. Two days post-challenge, mice were euthanased and blood and spleen samples collected for bacterial-burden determination. The antisera from CRM or PBS immunised mice was used as control.

### Data availability Statement

All data generated or analysed during this study are included in this published article (and its Supplementary Information files).

## Electronic supplementary material

Supplementary Information
