# Supplementary material for: Physicochemical characterisation, immunogenicity and protective efficacy of a lead streptococcal vaccine: progress towards Phase I trial
Source: Sci Rep. 2017 Oct 23;7:13786. doi: 10.1038/s41598-017-14157-7 (PMC5653875; doi:10.1038/s41598-017-14157-7)
Supplement: Supplementary file 1 — Supplementary Information [file 41598_2017_14157_MOESM1_ESM.pdf]

Physicochemical characterisation, immunogenicity and protective efficacy of a lead streptococcal vaccine: progress towards Phase I trial

Manisha Pandey*, Jessica Powell, Ainslie Calcutt, Mehfuz Zaman, Zachary N Phillips, Mei Fong Ho, Michael R Batzloff and Michael F Good*

Institute for Glycomics, Griffith University, Queensland 4222, Australia.

Supplementary Table1. Hydropathy index of S2 and its derivatives with flanking Lysine residues

Name	Sequence	Hydropathy index*
S2	NSDNIKENQFEDFDEDWENFC	-1.70
KKS2KK (K2S2K2)	KKNSDNIKENQFEDFDEDWENFKKC	-2.05
KKKKS2 (K4S2)	KKKKNSDNIKENQFEDFDEDWENFC	-2.05
S2KKKK (S2K4)	NSDNIKENQFEDFDEDWENFKKKKC	-2.05

*Hydropathy index analysed using protein bioinformatics tool GPMAW lite.

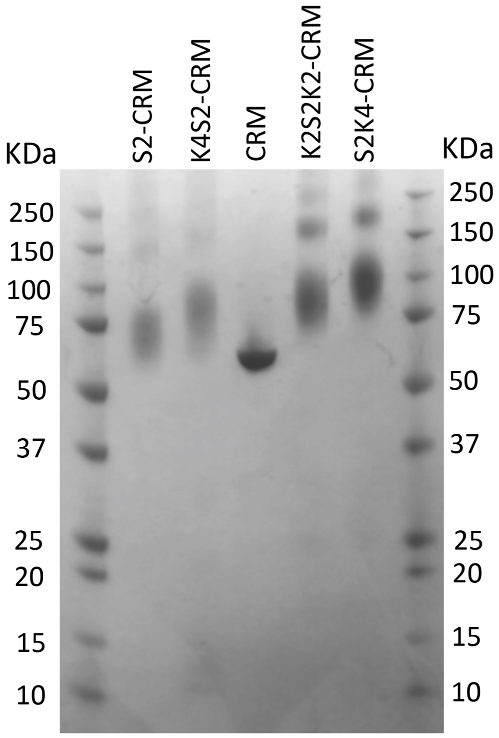

Sup fig 1. SDS-PAGE profile of S2 derivatives-CRM197 conjugates. (A) To enhance the solubility of SpyCEP epitope S2 in aqueous solution, lysine residues were added to S2 at either N-or C-termini, or at both ends. The resulting three S2 derivatives, as well as the parent S2 peptide, were conjugated to CRM197 (CRM) and characterised by SDS-PAGE. CRM alone was used as a control. The protein conjugates were resolved by poly acrylamide gels and visualised using commassie blue stain. A full-length image of gel in Fig 1A is presented.

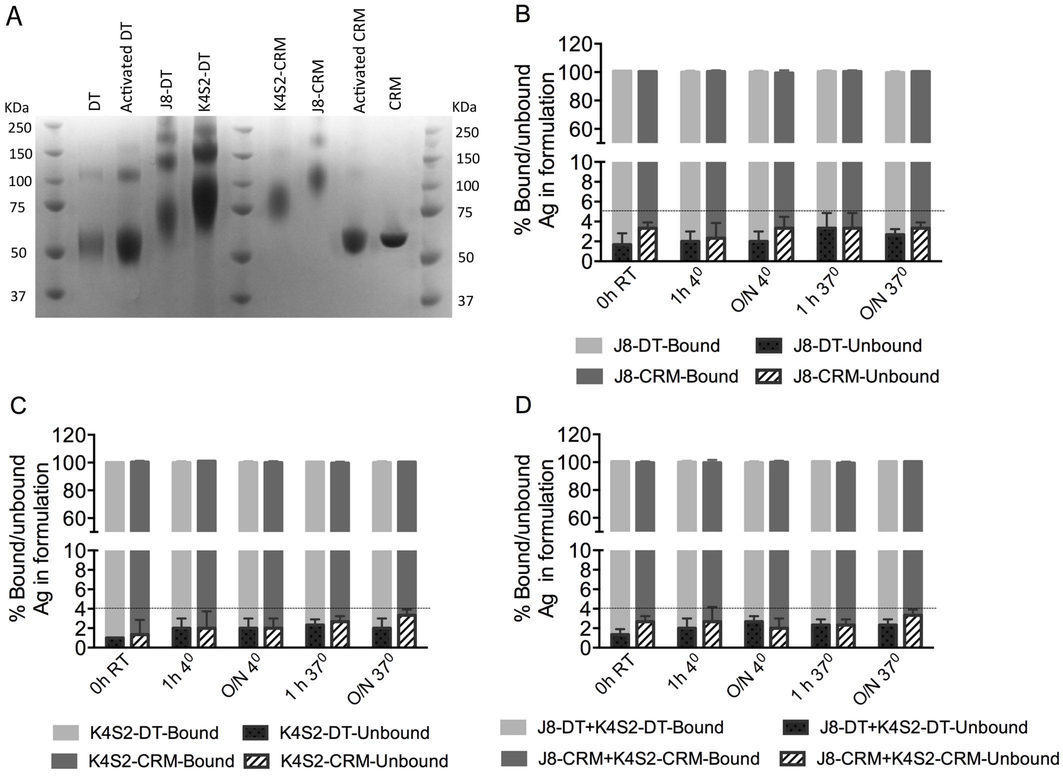

Sup fig 2. Characterisation of CRM conjugates. (A) A SDS-PAGE profile comparing the DT- and CRM- conjugated vaccine components. The relative molecular mass of conjugates were determined using SDS-PAGE. The proteins were resolved by 4-15% poly acrylamide gels and visualised using Coomassie blue stain. (B-D) **Comparative adsorption of DT- and CRM- conjugates onto Alum**. To compare the adsorption of DT- and CRM- conjugated peptides onto Alum, adsorption assays were undertaken. Various DT- and CRM- conjugated peptides (J8, K4S2 and combination) were added to a known volume of Alum and incubated for varying lengths of time at a range of temperature conditions. The percent bound and unbound peptides in the conjugate-Alum formulation at 0h RT, 1h 4^0C, O/N 4^0C, 1h 37^0C and O/N 37^0C for J8 (B), K4S2 (C) and combination J8+K4S2 (D) are shown. Data for each bar are mean ±SEM. The dotted line at the bottom denotes the maximum amount of free peptides in the formulation.

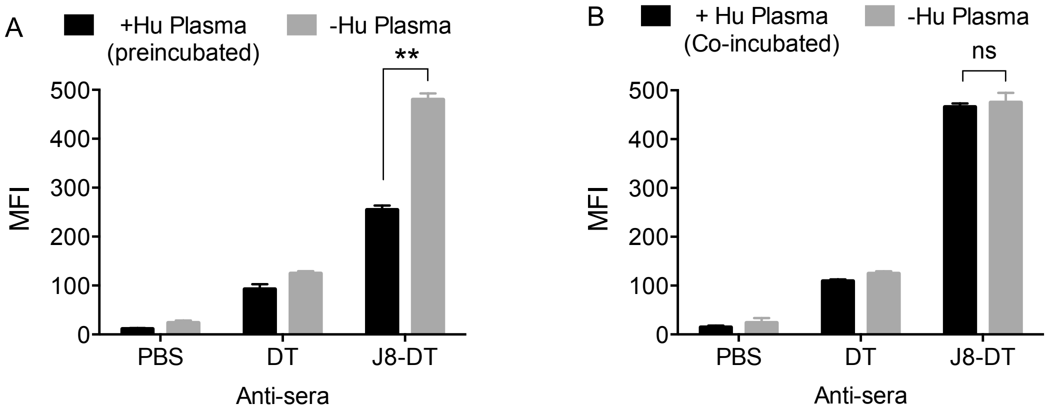

Sup fig 3. Binding of vaccine antibodies to the bacterial surface in the presence of human plasma. To assess the effect on human plasma on binding of vaccine antibodies to the bacterial surface, flow cytometry was performed. The non-specific binding sites on the surface of 2031 (*emm1*) GAS were blocked with Fc blocker (2.4G2 mAb). Following incubation with normal human plasma or PBS, vaccine/control antisera were added to the mix (A). In parallel, following non-specific blocking, GAS were co-incubated, in a ratio of 1:1 with vaccine/control antisera in the presence or absence of human plasma (B). After 1h incubation at 4^0C, the bacteria were washed twice in PBS followed by incubation with a FITC-conjugated anti-mouse IgG (diluted 1/50 in PBS with 2% BSA) for another 30 minutes at RT. The bacteria were washed and incubated in 300 µl of 1 % formaldehyde (in PBS) for 15 minutes at RT and then transferred to ice until reading on a FACS Calibur Flow Cytometer (Becton Dickinson, USA). The antisera from PBS immunised mice was used as control. Data for each bar are mean ±SEM. Significance between the binding efficiency of test and control antisera in the presence or absence of human plasma was determined using a Student's t-test. ns >0.05; **p<0.01.
